# NADPH Oxidase *versus* Mitochondria-Derived ROS in Glucose-Induced Apoptosis of Pericytes in Early Diabetic Retinopathy

**DOI:** 10.1155/2010/746978

**Published:** 2010-06-28

**Authors:** Nik M. Mustapha, Joanna M. Tarr, Eva M. Kohner, Rakesh Chibber

**Affiliations:** ^1^Forest Research Institute Malaysia (FRIM), 52109 Kepong, Selangor Darul Ehsan, Malaysia; ^2^Cardiovascular Division, GKT School of Biomedical & Health Sciences, King's College London, Guy's Campus, London SE1 1UL, UK; ^3^Institute of Biomedical and Clinical Science, Peninsula College of Medicine and Dentistry, Peninsula Medical School, St Luke's Campus, Exeter EX1 2LU, UK

## Abstract

*Objectives*. Using apocynin (inhibitor of NADPH oxidase), and Mitoquinol 10 nitrate (MitoQ; mitochondrial-targeted antioxidant), we addressed the importance of mitochondria versus NADPH oxidase-derived ROS in glucose-induced apoptosis of pericytes. *Methods*. NADPH oxidase was localised using Western blot analysis and cytochrome C reduction assay. Apoptosis was detected by measuring caspase-3 activity. Intracellular glucose concentration, ROS formation and N*ε*-(carboxymethyl) lysine (CML) content were measured using Amplex Red assay kit, dihydroethidium (DHE), and competitive immunoabsorbant enzyme-linked assay (ELISA), respectively. *Results*. NADPH oxidase was localised in the cytoplasm of pericytes suggesting ROS production within intracellular compartments. High glucose (25 mM) significantly increased apoptosis, intracellular glucose concentration, and CML content. Apoptosis was associated with increased gp91phox expression, activity of NADPH oxidase, and intracellular ROS production. Apocynin and not MitoQ significantly blunted the generation of ROS, formation of intracellular CML and apoptosis. *Conclusions*. NADPH oxidase and not mitochondria-derived ROS is responsible for the accelerated apoptosis of pericytes in diabetic retinopathy.

## 1. Introduction

Diabetic retinopathy is a leading cause of blindness that is characterized by vascular changes of the retinal capillary bed [1]. One of earliest changes is the accelerated apoptosis of retinal microvascular cells and the formation of acellular capillaries [2]. Although, the frequency of pericytes and endothelial cell apoptosis is thought to predict the development of the histological lesions in retinopathy [3], the underlying cause is not fully understood. Diabetes Control and Complications Trial (DCCT) and the United Kingdom Prospective Diabetes Study (UKPDS) have confirmed that hyperglycaemia is the major factor in the development of diabetic retinopathy [4, 5]. In addition to the prevailing biochemical mechanisms [1] on how glucose leads to pathological changes in retinopathy, recent evidence suggests a key role for oxidative stress, a state in which excess reactive oxygen species (ROS) overwhelm endogenous antioxidant systems [6]. ROS can be produced from the mitochondrial transport chain and a number of enzymes that are localised in the plasma membrane, and the cytoplasm of cells [7]. 

The elegant study carried out by Brownlee and colleagues [8] suggests that glucose-induced mitochondria production of ROS stimulates several of the biochemical mechanism thought to be involved in hyperglycaemia-mediated complications of diabetes, including retinopathy. The authors proposed that the causal link between glucose and vascular damage in diabetes is the increased production of superoxide by the mitochondrial electron transport chain [8]. However, increasing evidence suggests that NADPH oxidase is the most important source of cellular ROS in blood vessels [9]. NADPH oxidase complex in neutrophils and, most probably, endothelial cells and other cell types, involves four essential subunits. The subunits gp91phox and p22phox reside in the plasma membrane [9]. These subunits bind the components of the electron transport chain heme and FAD, forming cytochrome *b*_558_. The cytosolic NADPH oxidase subunits p47phox and p67phox are involved in the activation of the enzyme complex. Unlike the phagocytic type, the NADPH oxidases present in blood vessels are constitutively active, producing relatively low levels of ROS under basal conditions, and generating higher levels of oxidants in response to cytokines [9]. Among the nonphagocytic cells examined so far, endothelial NADPH oxidase has been investigated more extensively [9]. 

By using apocynin and Mitoquinol 10 nitrate (MitoQ) we addressed the importance of mitochondria versus NADPH oxidase-derived ROS in glucose-induced apoptosis of cultured retinal capillary pericytes. Apocynin is a methoxy-substituted catechol that does not act as a ROS scavenger, but inhibits NADPH oxidase by impeding the assembly of p47phox and p67phox subunits within the membrane NADPH oxidase complex [10]. MitoQ which has an antioxidant ubiquinol moiety attached to a triphenylphosphonium cation by an aliphatic carbon chain is recently developed mitochondria-targeted antioxidants that selectively block mitochondrial oxidative damage [11]. The selective accumulation of MitoQ prevents mitochondrial oxidative damage far more effectively than untargeted antioxidants. It is not only accumulated by the mitochondria but also can be regenerated in its reduced form by mitochondrial respiratory chain [11]. Here we show for the first time, that it is ROS derived from NADPH oxidase and not the mitochondria, which is involved in apoptosis of pericytes induced by chronic exposure to high glucose.

## 2. Materials and Methods

### 2.1. Culture of Bovine Retinal Capillary Pericytes

Bovine retinal capillary pericytes (BRPs) were established from bovine retinas dissected from eyes of freshly slaughtered cattle as described previously in [12]. Briefly, the isolated retinas were homogenised in serum-free minimal essential medium (MEM; Sigma, UK), and filtered through 80 *μ*m nylon mesh. The trapped micro vessels were digested with collagenase-dispase (1 mg/ml) for 30 min at 37°C, filtered through a 45 *μ*m nylon mesh and then plated in tissue culture flasks and maintained in MEM supplemented with 10% foetal calf serum (FCS), 2 mM glutamine, 100 IU/ml penicillin, and 100 *μ*g/ml streptomycin. The cells were characterized as described previously [12] and used at passage 2-3.

For experiments, confluent cultures of BRP were exposed to continuous normal (5.6 mM) glucose and high (25 mM) glucose for 4 days. In some experiments, 500 *μ*M apocynin and 1 *μ*M of the mitochondrial targeted antioxidant [11], MitoQ (quinol attached to triphenylphosphonium; kindly provided by Dr Murphy, MRC-Dunn Human Nutrition Unit, Cambridge, UK) was added to normal and high-glucose medium.

### 2.2. Caspase-3-Like Activity

Cellular caspase-3 activity was determined using the colorimetric protease assay. This assay detects p-nitroanilide (p-NA) photometrically at 405 nm after its cleavage from the colorimetric substrate, N-acetyl-Asp-Glu-Val p-nitroanilide (Ac-DEVD-pNA) by the caspase-3 [13].

### 2.3. DNA Fragmentation

DNA fragmentation was quantified using the Cell Death Detection ELISA plus (1774425; Roche, Hertfordshire, UK). The assay was carried out according to manufacturer's instructions, which measures mono-, and oligonucleosomes in the cytoplasmic fraction of cell lysate. The assay was based on a quantitive sandwich enzyme-immunoassay directed against cytoplasmic histone-associated DNA fragments.

### 2.4. Western Blot Analysis

Antibodies directed against NADPH oxidase subunits, Gp91phox, and p47phox were from Upstate (UK). After treatment, cells were lysed on ice in the following lysis buffer (20 mM Tris-HCL, pH 7.4, 1% Triton X-100, 150 mM NaCl, 1 mM EDTA, 1 mM EGTA, 0.2 mM sodium vanadate, 1 mM PMSF, 1 *μ*g/ml aprotinin, 10 *μ*g/ml leupeptin). Equal amounts of protein were separated on 10% polyacrylamide gels and transferred to Hybond-ECL membranes (Amersham, UK). Membranes were blocked with 3% bovine serum albumin (BSA) in phosphate buffered saline (PBS) containing 0.05% Tween 20 (PBST). After immunodetection of p47phox, and gp91phox, the membranes were stripped in a buffer containing 50 mM Tris, 2% SDS, and 100 mM mercaptoethanol at 55°C for 30 min, washed and immunoblotted with tubulin antibody (Chemicon, Hampshire, UK). Immunoreactive bands were quantified by scanning densitometrically and calculating the density of individual bands using Image J software (National Institute of Health, Bethesda, Maryland, http://rsb.info.nih.gov/ij/). The levels of NADPH oxidase subunits were expressed as a ratio of intensity of p47phox, and gp91phox immunoreactive bands/intensity of tubulin immunoreactive bands.

### 2.5. Measurement of Intracellular ROS Production

The cell permeant dihydroethidium (DHE; Molecular Probes) was used to assess real-time formation of superoxide (O_2_^−^) in BRP exposed to normal (5.8 mM) and high (25 mM) glucose. DHE enters the cells and is oxidized by superoxide to form ethidium (ETH), which binds to DNA to produce the fluorescent ETH-DNA that displays red fluorescence [14]. DHE was prepared as 2 mM stock solution in DMSO and stored at −20°C. The cells were loaded with 10 *μ*M for the final 2 h of incubation. The cells were washed with PBS, lysed with 50 *μ*l lysis buffer (20 mM Tris-HCl, pH 7.4, 1% Triton X-100, 150 mM NaCl, 1 mM EDTA, 50 mM NaF and 0.2 mM sodium orthovanadate) on ice. The lysates were then transferred into black 96-well plates (Fisher, Loughborough, UK) and the fluorescence was measured using spectrofluorometer (Plate Chameleon; Hidex, Baringstoke, UK). ETH-DNA red fluorescence was measured with excitation at 530 nm and emission at 616 nm. Background fluorescence intensity was subtracted from the results and the level of ROS was expressed as fluorescence intensity/*μ*g protein.

### 2.6. Measurement of NADPH Oxidase Activity

Measurement of O_2_^−^ was based on the capacity to reduce ferricytochrome *c* in ferrocytochrome at pH 7.8 [15]. Total cell lysate (50 *μ*g protein/experiment), cytochrome c (250 *μ*g/l final concentration), and NADPH (100 *μ*M) were incubated at 37°C for 120 min, either in the presence or absence of diphenyleneiodonium (DPI, 100 *μ*M). The reduction of cytochrome c was measured by reading absorbance at 550 nm. O_2_^−^ production in nmol/mg protein was calculated from the difference between absorbance of samples at 0 and 120 min and the extinction coefficient 21 mmol/l/cm.

### 2.7. Measurement of Intracellular CML

The intracellular formation of N^*ε*^-(carboxymethyl) lysine (CML) in pericytes was quantified using competitive imuunoabsorbant enzyme-linked assay (ELISA) as previously described in [16]. CML-BSA, used as a standard, was dissolved in 0.05 M carbonate buffer, pH 9.6, to a concentration of 0.5 *μ*g/ml. A 50 *μ*l aliquot was added to each well of a 96-well microtitre ELISA plate (Nunc, Loughborough, UK). After incubation at 4°C overnight, the coating solution was discarded and the wells washed three times with 400 *μ*l of PBS containing 0.05% Tween-20 (PBST). The wells were then filled with 100 *μ*l of 2% normal goat serum (NGS) containing 0.1% BSA for blocking and left for 1 h at room temperature. After washing with three times with 400 *μ*l PBST, 50 *μ*l of the diluted standards and samples were added in triplicate wells followed by 50 *μ*l of the 1 : 1000 diluted CML-antibody (Kindly provided by Dr D Ruggiero, INSA, Lyon, France). After incubation for 2 h at room temperature, the wells were washed three times with 400 *μ*l PBST, and developed with goat antirabbit peroxidase-conjugated IgG (diluted 1 : 10,000 in PBST) and o-phenylenediamine (Sigma, Dorset, UK). The absorbance of the samples was read at 490 nm and the levels of CML determined using the standard curve prepared using various concentrations of CML-BSA. 

### 2.8. Measurement of Intracellular Glucose Concentration

After treatment, cells were washed and medium was removed and cells lysed. Intracellular glucose concentration was then determined using Amplex Red Glucose Assay Kit (A 22189; Molecular Probes, Paisley, UK) according to the manufacturer's instructions. Briefly, 50 *μ*l of the reaction solution (10 mM Amplex Red, 10 U/ml HRP, 100 U/ml glucose oxidase, 50 mM sodium phosphate buffer, pH 7.4) was added to 50 *μ*l of cell lysate in 96-well microtitre plate and incubated in the dark for 45 min at room temperature. The absorbance was then measured at 560 nm using a SpectraMax 190 microplate reader. Intracellular glucose concentration was determined from a standard curve generated using various concentrations of glucose.

### 2.9. Measurement of Protein Kinase C*β*1/2 Activity

Cellular PKC*β*1/2 activity was determined using the TruLight assay kit (Calbiochem, Nottingham, UK) according to the manufacturer's instructions. The activity was based on fluorescence superquenching and the assay was carried out in a white 96-well microtitre plates (Nunc, Loughborough, UK). Fluorescence was measured using microplate reader (Plate Chameleon, Hidex, Basingstoke, UK) with excitation at 450 nm and emission at 535 nm. PKC*β*1/2 activity was calculated from the generated phosphopeptide calibrator curve and the results expressed PKC*β*1/2-dependent phosphorylation/mg protein. 

### 2.10. Protein Measurement

Total protein was measured using the BCA protein assay kit (Pierce, UK). 

### 2.11. Statistical Analysis

The statistical software Graph Pad Prism version 3.0 was used. A two-tailed Student's *t*-test was used to test the significance of paired data. Data are expressed as means ± S.E.M of measurements in the different experiments. Differences between groups were considered statistically significant at *P* < .05.

## 3. Results

### 3.1. Retinal Capillary Pericytes Express NADPH Oxidase

**Figure 1:**
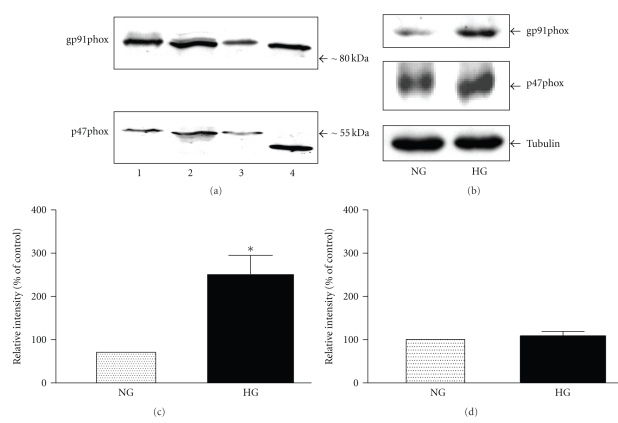
*Retinal capillary pericytes express NADPH oxidase*. Aliquots of total cellular protein were separated by SDS-PAGE, electrophoretically transferred onto Hybond-ECL membranes. Blots were probed with (a) anti-gp91phox antibody and anti-p47phox antibody. Protein antibody complexes were detected using secondary antibody conjugated with horseradish peroxidase and the ECL detection system. Lane 1: BRP, lane 2: BREC (bovine retinal capillary endothelial cells), Lane 3: HUVEC (human umbilical vein endothelial cells), and lane 4: U937 cells (human leukocytes). (b) Representative Western blots of gp91phox, p47phox and tubulin. (c) Expression of gp91phox protein expression in BRP exposed to high glucose (HG, 25 mM) for 48 h. Densitometric ratio (intensity gp91phox immunoreactive band/intensity of the tubulin immunoreactive band) is expressed as % of normal glucose (NG, 5.6 mM). (d) Expression of p47phox protein expression in BRP exposed to high glucose (HG, 25 mM) for 48 h. Densitometric ratio (intensity of p47phox immunoreactive band/intensity of the tubulin immunoreactive band) is expressed as % of normal glucose (NG, 5.6 mM). Data are represented as Means ± SEM of 3-4 separate experiments. *P < .05 versus control.

NADPH oxidase is a membrane-bound enzyme complex that is a source of cytosolic O_2_^−^ and is composed of at least four subunits, including two important membrane-bound subunits p22phox and gp91phox and two cytosolic subunits p47phox and p67phox. On a Western blot, the antibody directed against the N-terminal peptide labelled specifically only one broad band at approximately 80 kDa in lysates of BRP (Figure 1(a)). This immunoreactive band was detected using the antibody directed against the peptide corresponding to amino acids 548–560 of human gp91phox. The antibody also detected similar molecular weight band in cultured bovine retinal capillary endothelial cells (BREC), human umbilical endothelial cells (HUVEC), and human leukocytes (U937 cells) (Figure 1(a)). The anti-gp91phox antibody used in our study is specific to human gp91phox (Nox2) and corresponds to amino acids 548–560 of human Nox2. 

Specific anti-p47phox also confirmed the presence of p47phox NADPH oxidase subunit with approximate molecular weight of 55 kDa in BRP. An immunoreactive band of similar weight was also localized in BREC, and HUVEC (Figure 1(a)). However, in U937 cells the p47phox immunoreactive band had a lower molecular weight of approximately 45 kDa. Thus, two major components of a functional NADPH oxidase are present in BRP. 

Immunoblot analysis showed that 48 h exposure to high glucose significantly increased gp91phox protein expression (Figures 1(b) and 1(c)) to 249 ± 38% of normal glucose (*n* = 5, *P* < .05). In contrast, high glucose failed to cause a significant change in the expression of p47phox (Figures 1(b) and 1(d)).

### 3.2. Glucose Increases NADPH Oxidase Activity

**Figure 2:**
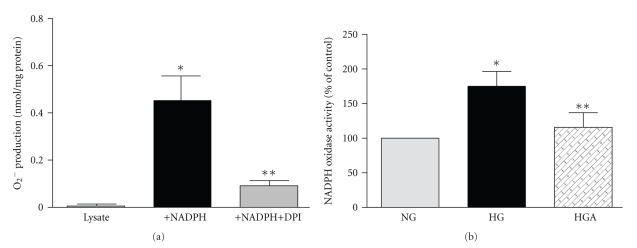
*Apocynin reverses glucose-induced NADPH oxidase activity*. (a) Cultured bovine retinal capillary pericytes express active NADPH oxidase. The activity was determined by cytochrome c reduction. Lysate (25 *μ*g protein) was added to 250 *μ*M cytochrome c in a 96-well multiwell plate and incubated for 120 min, either in the presence or absence of NADPH (100 *μ*M) or NADPH oxidase inhibitor or flavoprotein inhibitor, diphenyleneiodonium (DPI, 100 *μ*M). The reduction of cytochrome c was measured by reading absorbance at 550 nm (SpectraMax 190). Superoxide (O_2_^−^) production in nmol/mg protein was calculated from absorbance of samples and the extinction coefficient for change of ferricytochrome c to ferrocytochrome c of 21 mmol/l/cm. Data are presented as mean ± SEM of 4–6 separate experiments. *P < .001 versus lysate alone, **P < .01 versus lysate with NADPH. (b) Exposure to high glucose for 4 days increases NADPH oxidase which is significantly reversed by the addition of apocynin. Confluent cultures of BRP in 30 mm^2^ dishes were exposed to normal glucose (NG, 5.6 mM) and high glucose (HG, 25 mM) with and without 500 *μ*M apocynin (HGA) for 4 days. After incubation, the cells were washed twice with ice-cold PBS; lysed, and NADPH-dependent O_2_^−^ production was measured by DPI-inhibitable cytochrome c reduction assay. Data are presented as mean ± SEM of 4 separate experiments. *P < .05 versus HG. **P < .013 versus HG.

To provide further and more direct evidence for the effect of high glucose on NADPH oxidase, cellular activity was measured using the cytochrome c reduction assay. BRP expresses active NADPH oxidase (Figure 2(a)). High glucose increased the NADPH-induced O_2_^−^ generation by almost 1.65-fold compared to normal glucose (Figure 2(b)). The addition of apocynin (500 *μ*M) significantly inhibited glucose-induced NADPH oxidase activity (115 ± 22% of normal glucose, *n* = 4, *P* = .013).

### 3.3. Glucose Stimulates ROS in Pericytes

**Figure 3:**
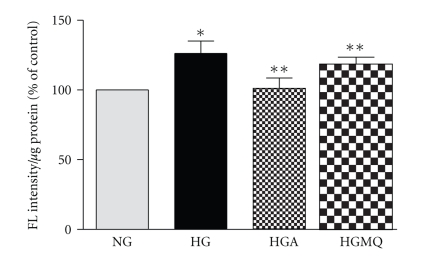
*Apocynin reverses continuous high glucose-induced ROS production in BRP*. Subconfluent cells in 24-well plate were exposed to normal glucose (NG, 5.6 mM) and continuous high glucose (HG, 25 mM) in the absence and presence of 500 *μ*M apocynin (HGA) or 1 *μ*M MitoQ (HGMQ) for 3 days. Approximately, 2 h before the end of incubation, DHE (10 *μ*M) was added. After the treatment, the cells were washed twice with PBS, lysed and fluorescence (FL) intensity was measured. Data are presented as mean ± SEM of 6 separate experiments. *P < .05 versus NG, **P < .05 versus HG.

Exposure to high glucose significantly increased the intracellular O_2_^−^ production as detected by ETH-DNA fluorescence (Figure 3). Oxidant generation increased 126 ± 9% of normal glucose (*n* = 6, *P* < .05) and was significantly reversed by treatment with 500 *μ*M apocynin (101 ± 7% of normal glucose versus 126 ± 9% of normal glucose, *n* = 6, *P* < .05). MitoQ slightly reversed glucose-induced ROS production (122 ± 4% of normal glucose, *n* = 6, *P* = .05).

### 3.4. Apocynin and Not MitoQ Reverse Glucose-Induced Apoptosis

**Figure 4:**
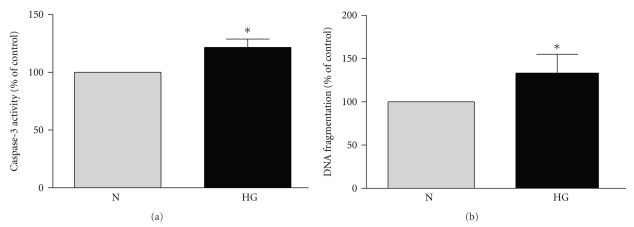
*Continuous high (25 mM) glucose induces apoptosis of BRP*. Subconfluent cells in 30 mm^2^ dishes were exposed to normal (N, 5.6 mM) and high (CG, 25 mM) glucose for 4 days. (a) High glucose activates caspase-3. After 4 days, the cells were washed twice with ice-cold PBS, lysed and apoptosis was measured using colorimetric caspase activity assay. Data are represented as Means ± SEM of 6 separate experiments. *P = .0315 versus N. (b) High glucose induces DNA fragmentation. After 4 days, DNA fragmentation was measured using Cell Death Detection ELISA plus (Roche, Hertfordshire, UK). Data are represented as Means ± SEM of 5 separate experiments. *P* = .043 versus N.

Figure 4 compares the effect of high glucose on apoptosis determined by measuring cellular caspase-3 activity (A) and the level of DNA fragmentation (B) using the Cell Death Detection ELISA Plus (Roche, Hertfordshire, UK) which measures mono- and oligonucleosomes in the cell lysate. Both assay confirmed high glucose-induced apoptosis of BRP after 4 days. 

**Figure 5:**
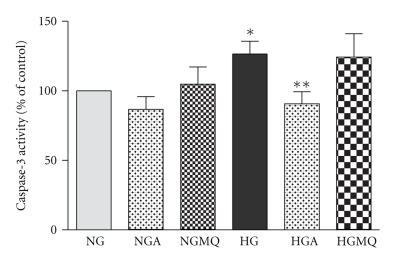
*Chronic exposure to high glucose induces apoptosis in BRP.* Subconfluent cells in 3 cm dishes were exposed to normal (NG, 5.6 mM) and high (HG, 25 mM) glucose in the absence (NG, HG) and presence of 500 *μ*M apocynin (NGA, HGA) or 1 *μ*M MitoQ (NGMQ and HGMQ). After 4 days, the cells were washed twice with ice-cold PBS, lysed and apoptosis was measured using colorimetric caspase-3 activity. Data are represented as Means ± SEM of 3–6 separate experiments. *P < .05 versus NG, **P < .05 versus HG.

 As shown in Figure 5, incubation for 4 days in continuous high glucose caused a significant increase in apoptosis of pericytes compared to normal glucose (127 ± 9% of normal glucose, *n* = 7, *P* < .001). This glucose-induced caspase-3 activity was significantly reversed (Figure 5) by 500 *μ*M apocynin (91 ± 7% of normal glucose versus 127 ± 9% of normal glucose, *n* = 6 − 13, *P* < .05). In contrast, the addition of MitoQ failed to prevent glucose-induced activation of caspase-3 (124 ± 17% of normal glucose, *n* = 4).

### 3.5. Intracellular CML Content after Exposure to High Glucose

**Figure 6:**
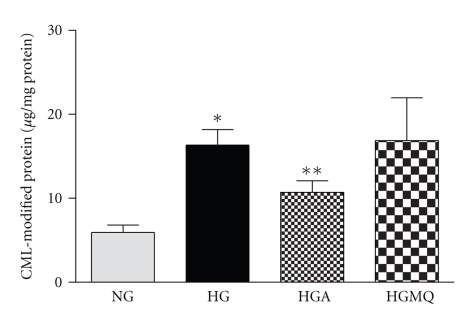
*Apocynin reverses glucose-induced CML formation in BRP*. Subconfluent cells in 24-well plate were exposed to normal glucose (NG, 5.6 mM) and continuous high glucose (HG, 25 mM) and in the presence of 500 *μ*M apocynin (HGA) or 1 *μ*M MitoQ (HGMQ) for 4 days. After incubation, the cells were washed twice with ice-cold PBS, lysed and CML were detected using competitive ELISA. Data are presented as Mean ± SEM of 8–13 samples. *P < .05 versus NG, **P < .05 versus HG.

Intracellular levels of CML were quantified by competitive enzyme-linked immunoabsorbant assay (ELISA) using specific CML antibody. Standard curve was established using various concentration of CML-BSA. As shown in Figure 6, exposure to continuous high glucose for 4 days significantly increased the intracellular CML content by almost 2.8-fold compared to normal glucose (16.3 ± 1.9 *μ*g/mg protein versus 5.9 ± 0.9 *μ*g/mg protein in normal glucose, *n* = 10–12, *P* < .05). The addition of apocynin at 500 *μ*M, a concentration that reverses glucose-induced apoptosis, significantly prevented the accumulation of CML in pericytes exposed to high glucose (10.7 ± 1.4 *μ*g/mg protein versus 16.3 ± 1.9 *μ*g/mg protein in high glucose, *n* = 8–12, *P* < .05). In contrast, MitoQ, (1 *μ*M) had no significant effect on the CML content in pericytes exposed to high glucose (16.9 ± 5.1 *μ*g/mg protein, *n* = 8).

### 3.6. Glucose-Induced PKC-*β* Activity

There was no significant increase in PKC-*β*1/*β*2 activity measured using fluorescence superquenching-based assay. After 4 days exposure, the activity expressed as PKC-*β*1/*β*2-dependent phosphorylation/mg protein was found to be 6.9 ± 3.5 (*n* = 6) in HG compared to 6.8 ± 3.5 (*n* = 6).

### 3.7. Increased Accumulation of Intracellular Glucose after Exposure to High Glucose

**Figure 7:**
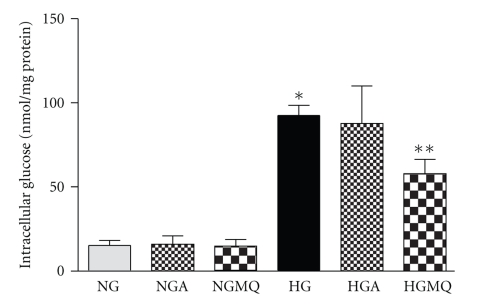
*Exposure to high glucose increases intracellular glucose concentration*. Subconfluent cells in 24-well plate were exposed to normal glucose (NG, 5.6 mM) and high glucose (HG, 25 mM) and in the presence of 500 *μ*M apocynin (NGA, HGA) or 1 *μ*M MitoQ (NGMQ, HGMQ) for 4 days. The cells were washed twice with ice-cold PBS, lysed and intracellular glucose concentrations were measured using the Amplex Red glucose assay kit (Molecular Probe). Data are Means ± SEM of 5–14 separate experiments. *P < .001 versus NG; **P < .05 versus HG.

Continuous exposure to high glucose (25 mM) for 4 days increased the intracellular glucose concentration by almost 6-fold compared to BRP exposed to normal glucose (92.6 ± 6.0 nmol/mg protein versus 15.2 ± 3.0 nmol/mg protein in normal glucose, *n* = 13-14, *P* < .001) (Figure 7). At 500 *μ*M, apocynin failed to prevent the accumulation of intracellular glucose (87.8 ± 22.3 nmol/mg protein, *n* = 5). Interestingly, although MitoQ (1 *μ*M) failed to prevent the activation of caspase-3, it significantly lowered the level of intracellular glucose concentration in BRP exposed to high glucose (57.9 ± 8.6 nmol/mg protein versus 92.6 ± 6.0 nmol/mg protein in high glucose, *n* = 5–13, *P* < .05). However, apocynin and MitoQ did not alter the intracellular glucose concentration in BRP exposed to normal glucose.

## 4. Discussion

There is now growing evidence that oxidative stress plays an important role in the pathogenesis of chronic complications of diabetes [6], but the exact source, and cellular location of the glucose-induced ROS is still unclear. Brownlee and co-workers proposed that in cultured macrovascular bovine aortic endothelial cells (BAECs), the production of ROS by the mitochondria *via* the respiratory chain is the most important causal link between high glucose and the main pathways responsible for hyperglycaemic damage [8]. Besides mitochondria, NADPH oxidase also generates a significant amount of ROS and is a major source of superoxide in vascular cells [9]. In the present study, we used apocynin, an inhibitor of NADPH oxidase [10] and MitoQ, a mitochondria-targeted antioxidant [11, 12], to explore the importance of mitochondria versus NADPH oxidase derived ROS in glucose-induced apoptosis of cultured retinal capillary pericytes. 

Our observations of glucose-induced apoptosis of pericytes is consistent with previous *in vitro* studies [17–20] and the activation of caspase-3 in the retina of diabetic animals and humans [21, 22]. Consistent with recent reports [23, 24], retinal capillary pericytes express NADPH oxidase as indicated by the immunoblotting of Nox2 and p47phox, major membrane and cytosolic subunits [9, 10], respectively. In contrast, Manea et al. [24] using reverse transcriptase-polymerase chain reaction (RT-PCR) detected Nox1 and Nox2 in pericytes isolated from rat adipose tissue microvasculature. Since Nox2 and Nox1 shows only 56% of homology [25], we safely assume that our antibody does not cross-react with Nox1. NADPH oxidase, as in other cell types [26, 27] could be mostly present in the cytoplasm of pericytes, suggesting that ROS is produced within the intracellular compartments. Although, glucose increased NADPH oxidase *via *Nox2 expression, the exact mechanism of control is at present unclear. In support of our observations, increased mRNA and/or protein levels of gp91phox have been reported in blood vessels from animals [28, 29] and patients with diabetes [30]. As reported in other cell types [22, 31, 32], exposure to high glucose increased NADPH oxidase activity and ROS production in pericytes. In addition to changes in the expression of NADPH oxidase subunits [33, 34], high glucose could stimulate ROS production through protein kinase C (PKC)-dependent phosphorylation of the p47phox subunit [32]. The *β* isoform of PKC has been implicated in the phosphorylation of p47phox [33], but in our study, we failed to demonstrate activation of PKC*β*1/2 in pericytes exposed to high glucose. 

Our findings of increased oxidative stress in response to high glucose is in line with some previous studies [20, 23], but it appears to argue against a recent report suggesting that pericytes are resistant to glucose-induced oxidative stress [35]. The reason for this inconsistency is at present unclear. Our results show that ROS derived from NADPH oxidase and not the mitochondria, is involved in caspase-3-mediated apoptosis of pericytes induced by high glucose. This possibility is supported by recent studies [36] demonstrating the potential of apocynin to block glucose-induced ROS in BAEC. Other workers have demonstrated the role of NADPH oxidase in diabetic retinopathy [36–39], and complications of diabetes [40, 41] including the loss of podocytes in diabetic nephropathy [42]. Results with MitoQ suggested that some ROS is produced from the mitochondria, but it does not play a significant role in glucose-induced apoptosis. The mitochondria is known to play a key role in activating apoptosis through enhanced cytochrome (cyt c) release resulting in the activation of caspases and subsequent cell death [43]. Apoptotic cell death is mediated by stimulated caspase-3 activity, and the accumulation of p53, a known signalling molecule that acts upstream of caspase-3. Although, there is no direct evidence available to show that ROS derived from NADPH oxidase interact with mitochondria, we suggest that there is some interaction in pericytes exposed to high glucose. 

In bovine aortic endothelial cells (BAECs) the mitochondria-derived ROS was also involved in glucose-induced intracellular AGE formation [6]. However, our observation that apocynin reverses glucose-induced N^*ε*^-(carboxymethyl) lysine (CML) production, suggests a role of NADPH oxidase-derived ROS in the formation of intracellular CML-modified proteins in pericytes exposed to high glucose. In support of this, there is evidence that phagocytic NADPH oxidase plays an important role in CML formation *in vivo* [44]. Since CML is a biomarker of cellular oxidative stress [45], it may explain our previous failure to observe intracellular formation of AGEs, as detected using AGE-antibody [16] in pericytes exposed to high glucose. As reported previously [16, 46, 47] intracellular glucose levels were significantly increased in pericytes exposed to high glucose. During the initial step of auto-oxidative glycation, ROS fragments glucose to generate glyoxal with potential to react and form CML on lysine residues of intracellular and extracellular proteins [48]. The formation of CML from intermediates of the Mailard reaction, such as Schiff's base and the Amadori product that are raised in pericytes exposed to high glucose [16], requires ROS-mediated oxidative cleavage of the carbon backbone [49]. Although, ROS controls the formation of intracellular CML, its role in glucose-induced apoptosis is unclear, but may be involved in the observed reduced proliferation and increased necrosis of pericytes in high glucose. This notion is supported by a recent study showing that CML-modification of histones is associated with decreased proliferation of keratinocytes exposed to glyoxal [50]. Intracellular formation of CML in pericytes could well be important since increased levels of CML are present in the retina of rats with experimental diabetes [51] and levels in lymphocytes are associated with the pathogenesis of diabetic retinopathy [52]. 

In summary, we have demonstrated for the first time that in contrast to BAEC, ROS from NADPH oxidase and not the mitochondria plays a key role in glucose-induced intracellular formation of CML and apoptosis of retinal capillary pericytes. The results support the recent reports linking NADPH oxidase and diabetic retinopathy [39] and the beneficial effect of antioxidants to prevent oxidative stress and caspase-3-dependent apoptosis of pericytes [19]. Specific pharmacological NADPH oxidase inhibitors might also be of potential use during the treatment of early diabetic retinopathy.

## Nonstandard Abbreviations

ROS: Reactive oxygen species
BRP: Bovine retinal capillary pericytes
BAEC: Bovine aortic endothelial cells
HUVEC: Human umbilical vein endothelial cells
Ac-DEVD-pNA: N-acetyl-Asp-Glu-Val p-nitroanilide
p-NA: p-nitroaniline
FCS: Foetal calf serum
PBS: Phosphate buffered saline
OH: Hydroxyl radical
H_2_O_2_: Hydrogen peroxide
O_2_^−^: superoxide radical
BSA: Bovine serum albumin
DPI: diphenyleneiodonium
SOD: Superoxide dismutase
DHE: Dihydroethidium hydroethidine
ETH: Ethidium
SDS-PAGE: Sodium dodecyl sulphate polyacrylamide gel electrophoresis
ELISA: Enzyme linked immunoabsorbant assay
S.E.M: Standard error of mean
FAD: Flavin adenine dinucleotide
MitoQ: 10-(6′-ubiquinonyl) decyltriphenylphosphonium
AGE: Advanced glycation product.
